# *AtAUEs*, a Small Family of ABA Up-Regulated EAR Motif-Containing Proteins Regulate ABA Responses in Arabidopsis

**DOI:** 10.3390/plants13233282

**Published:** 2024-11-22

**Authors:** Wei Wang, Xutong Wang, Xiaoyu Liu, Yating Wang, Yingying Li, Saddam Hussain, Xiaoxiao Jing, Siyu Chen, Shucai Wang

**Affiliations:** 1 Laboratory of Plant Molecular Genetics and Crop Gene Editing, School of Life Sciences, Linyi University, Linyi 276000, China; wangwei220201@163.com (W.W.); wangxutong0019@163.com (X.W.); 19862161073@163.com (X.L.); hase705@nenu.edu.cn (S.H.); jingxiaoxiao86@163.com (X.J.); 2 Heilongjiang Provincial Key Laboratory of Environmental Microbiology and Recycling of Argo-Waste in Cold Region, College of Life Science and Biotechnology, Heilongjiang Bayi Agricultural University, Daqing 163319, China; wangyt814@nenu.edu.cn; 3 Key Laboratory of Molecular Epigenetics of MOE, Northeast Normal University, Changchun 130024, China; liyy857@nenu.edu.cn

**Keywords:** *AtAUEs*, abscisic acid, EAR proteins, CRISPR/Cas9, transcription repressor, Arabidopsis

## Abstract

The abscisic acid (ABA) signaling pathway is crucial for regulating downstream ABA-responsive genes, which influence plant responses to ABA and abiotic stresses. However, many ABA-responsive genes remain poorly characterized. This study reports on the identification and characterization of ABA up-regulated EAR motif-containing proteins (*AtAUEs*), a novel family of EAR motif-containing proteins in *Arabidopsis thaliana*. From a previous transcriptome dataset, *AtAUEs* were identified as a family of unknown-function ABA-response genes with only five members, and the up-regulation of *AtAUEs* by ABA was further confirmed by quantitative RT-PCR (qRT PCR). All *AtAUEs* contain at least one LxLxL EAR motif and can repress reporter gene expression in Arabidopsis protoplasts. We generated CRISPR/Cas9 gene-edited *ataue1*, *ataue2* and *ataue3* single, *ataue1 ataue2* (*ataue12*) double, and *ataue1 ataue2 ataue3* (*ataue123*) triple mutants, as well as transgenic plants overexpressing *AtAUE1*, and examined their ABA sensitivity. We found that the single and double mutants displayed wild-type responses to ABA treatment, while the *ataue123* triple mutants showed increased sensitivity in seed germination and cotyledon greening assays but decreased sensitivity to ABA treatment in root elongation assays. Conversely, the *35S:AtAUE1* showed decreased sensitivity in seed germination and cotyledon greening assays but increased sensitivity to ABA treatment in root elongation assays. The qRT PCR results show that the expression level of *ABI5* was increased in the *ataue123* mutants and decreased in the *35S:AtAUE1* plants. These findings suggest that *AtAUEs* function redundantly to regulate ABA responses in Arabidopsis, likely by modulating the expression of key regulatory genes in ABA-signaling pathway.

## 1. Introduction

The plant hormone abscisic acid (ABA) regulates various aspects of plant growth and development, including senescence, seed germination, and seedling development [1,2]. Primarily, ABA is recognized as a key regulator of plant responses to abiotic stress [3,4,5], acting through signal transduction to influence downstream ABA-responsive gene expressions [4,6,7,8,9,10].

ABA signaling involves several key proteins: The receptor proteins, specifically the Pyrabactin Resistance (PYR)/PYR1-Like/Regulatory Components of Abscisic Acid (ABA) Receptors, collectively referred to as PYR/PYL/RCAR [11,12], negative regulators known as Type 2C Protein Phosphatases (PP2Cs) [13,14], positive regulators from the Snf1 (Sucrose-Non-Fermentation 1)-Related Kinases Subfamily 2 (SnRK2s) [15], and the downstream ABA-Responsive Element-Binding Protein/ABRE-Binding Factor/ABA Insensitive 5 (ABF/AREB/ABI5)-type basic leucine zipper (bZIP) transcription factors that play crucial roles in signaling pathways [16,17].

In the absence of ABA, PP2Cs bind to and inhibit SnRK2s. In contrast, in the presence of ABA, they bind to PYR/PYL/RCAR receptors, promoting their interaction with PP2Cs, which subsequently leads to the release and auto-activation of SnRK2s. Activated SnRK2s then phosphorylate ABF/AREB/ABI5-type bZIP transcription factors, regulating downstream ABA-responsive genes and enhancing plant responses to abiotic stress [3,4,7,9,17,18,19,20]. Key ABA-responsive genes in Arabidopsis include the bZIP transcription factor gene *AtbZIP62*, the APETALA2 (AP2) transcription factor gene *ABI4*, the B3 transcription factor gene *ABI3*, the R2R3 MYB transcription factor gene *MYB71*, and novel transcription repressor genes such as *ABA-Induced Transcription Repressors* (*AITRs*) and *ABA-induced Serine-rich Repressors* (*ASRs*) [10,21,22,23,24,25,26,27]. However, the functions of most ABA-response genes remain largely uncharacterized.

The amino acid signature (L/F)DLN(L/F)xP was initially identified as the ERF-associated amphiphilic repression (EAR) motif in class II Ethylene Responsive Factor (ERF) repressors [27]. This motif was subsequently refined to DLNxxP and LxLxL through analysis of class II ERF repressors, C2H2 transcription factor repressors, and other EAR motif-containing proteins. It is believed that transcriptional repression in plants is primarily mediated by EAR motif-containing proteins [28].

These proteins can repress downstream gene expressions either independently as transcription repressors or by recruiting co-repressor proteins [29,30,31,32,33,34,35,36,37]. Notably, EAR motif-containing proteins display significant diversity in their amino acid sequences [35], leading to varied functions in plants. For instance, Arabidopsis Ovate Family Protein 1 (OFP1) independently regulates cell elongation by inhibiting the gibberellin (GA) biosynthesis gene *GA20-oxidase 1* (*GA20ox1*) [38], while Arabidopsis Kinase-Inducible Domain Interacting 8 (KIX8) and KIX9 promote leaf growth by recruiting the co-repressor TOPLESS to suppress downstream gene expressions [39].

Over 400 EAR motif-containing proteins have been identified in Arabidopsis through genome-wide searches for the DLNxxP or LxLxL signatures, yet most remain functionally uncharacterized [35]. Given the high amino acid sequence diversity among these proteins [34] and the brevity of the EAR motifs, it is probable that there are still some EAR motif-containing proteins that remain undiscovered. Our work on ABA-response genes of unknown function has led to the discovery of several novel ABA-response regulators in Arabidopsis [10,23,26,37,40,41]. This includes ABA-Induced Transcription Repressors (AITRs), ABA-induced Serine-rich Repressors (ASRs), and *Arabidopsis thaliana* EAR motif-containing ABA up-regulated proteins (AtEAUs), which feature LxLxL type EAR motifs. Notably, some of these proteins were not recognized in prior genome-wide assessments [10,26,40].

In this study, we present the identification and detailed characterization of *Arabidopsis thaliana* ABA up-regulated EAR motif-containing proteins (*AtAUEs*), a small family of five members with previously unknown roles in ABA responses. We found that ABA up-regulates the expression of *AtAUEs*, which contain at least one LxLxL EAR motif and function as transcription repressors. Through CRISPR/Cas9 gene editing, we generated single, double, and triple mutants for *AtAUE1*, *AtAUE2*, and *AtAUE3*, as well as transgenic plants overexpressing *AtAUE1*, to assess their ABA responses. Our findings suggest that *AtAUEs* operate redundantly to regulate ABA responses in a tissue- or growth-stage-dependent manner, potentially influencing the expression of the key ABA-signaling regulator gene *ABI5*.

## 2. Results

### 2.1. *AtAUEs* with EAR Motif Are ABA Up-Regulated Genes

**Figure 1:**
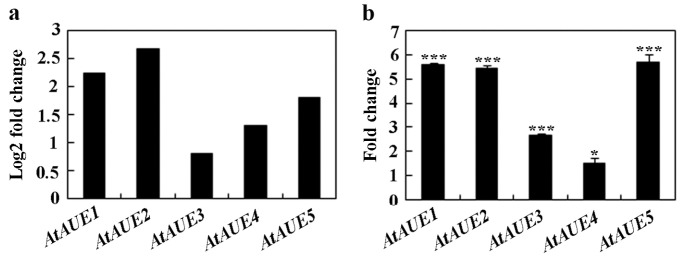
Induction of *AtAUEs* by ABA treatment. (**a**) *AtAUEs* are identified as ABA-responsive genes based on RNA-seq analysis. Log2 fold changes were calculated by comparing expression levels in ABA-treated versus mock-treated seedlings. (**b**) Fold changes in *AtAUEs* in response to ABA. Eight-day-old Col seedlings were treated with 50 mM ABA or methanol (mock) for 4 h. RNA was isolated, and cDNA was synthesized for quantitative Real-Time PCR (qRT-PCR) analysis. *ACT2* was used as the internal control. Data represent mean ± SD of three biological replicates. Significantly different from that of the mock-treated seedlings (* *p* < 0.05, *** *p* < 0.001).

In order to discover novel regulators involved in ABA and/or abiotic stress responses, we decided to identify and characterize unknown function ABA-response genes from a transcriptome dataset, as described previously [10]. During this process, we found that *At2g31940/AtAUE1* and its four closely related genes, i.e., *At5g19875/AtAUE2*, *At5g42146/AtAUE3*, *At3g06890/AtAUE4*, and *At2g21180/AtAUE5* are function uncharacterized ABA-response genes, with log2-fold changes ranging from ~0.8 to ~2.2 (Figure 1a). To further confirm that these genes are ABA-responsive genes, we examined their expression in Arabidopsis seedlings in response to ABA treatment. Col wild-type seedlings were subjected to either ABA or no ABA for a duration of 4 h, followed by the isolation of RNA, and then subjected to cDNA synthesis, and the cDNA synthesized was used for quantitative RT-PCR (qRT-PCR) to examine the expression levels of *AtAUEs*. As shown in Figure 1b, when compared to mock-treated seedlings, the expression levels of *AtAUEs* increased in ABA-treated seedlings, with a fold change ranging from ~1.5 to ~6, which is largely consistent with the data obtained from the transcriptome dataset.

**Figure 2:**
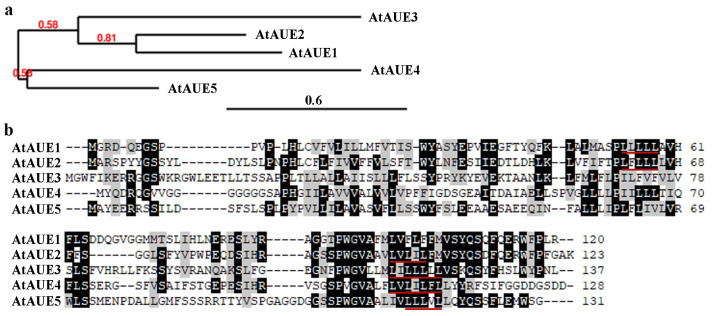
Phylogenetic analysis and amino acid sequence alignment of *AtAUEs*. (**a**) Phylogenetic analysis of *AtAUEs* was conducted using full-length amino acid sequences on the Phylogeny website (www.phylogeny.fr, accessed on 30 June 2024) with default settings in “One Click” mode. Branch support values are shown above the branches. (**b**) Amino acid sequence alignment of *AtAUEs* was performed using BioEdit. Identical amino acids are shaded in black, similar amino acids in gray, and LxLxL motifs are indicated by red underlines.

Phylogenetic analysis by using full-length amino acid sequences shows that AtAUE1 is closely related to AtAUE2 and formed one cluster of genes together with AtAUE3, whereas AtAUE4 and AtAUE5 formed another cluster of genes (Figure 2a). Amino acid sequence alignment results indicate that *AtAUEs* are predominantly conserved at their C-terminal regions, with all *AtAUEs* containing one LxLxL EAR motif, except for AtAUE2, which possesses two LxLxL EAR motifs (Figure 2b). Notably, the features described—specifically, the increased expression levels of the five genes in response to ABA treatment, along with the LxLxL EAR motif in the proteins they encode—are also evident in *Arab. Thaliana EAR motif-containing ABA up-regulated proteins 1* (*AtEAU1*) and *2* (*AtEAU2*) [36], but they are completely unrelated genes. To avoid any confusion about the five newly identified genes in this work, we named them *Arabidopsis thaliana ABA up-regulated EAR motif-containing 1* (*AtAUE1*) to *5* (*AtAUE5*).

### 2.2. *AtAUEs* Are Able to Repress the Expression of Co-Transfected Reporter Gene in Protoplasts

Given that EAR motif-containing proteins can mediate transcriptional repression [29] and all *AtAUEs* contain at least one LxLxL EAR motif, we investigated whether *AtAUEs* function as transcriptional repressors.

**Figure 3:**
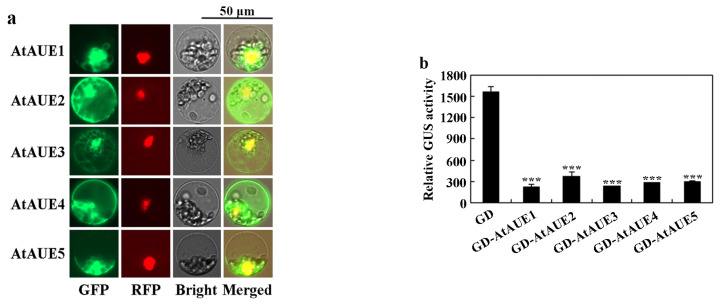
Subcellular localization and transcriptional activity of *AtAUEs*. (**a**) Subcellular localization of *AtAUEs*. Plasmids of *GFP-AtAUEs* were co-transfected with *NLS-RFP* into protoplasts isolated from Arabidopsis leaves. The transfected protoplasts were incubated in the dark for 22 h at room temperature, and GFP and RFP fluorescence was examined using a confocal microscope. (**b**) Transcriptional activity of *AtAUEs*. Plasmids of the *LexA-Gal4:GUS* reporter, *LD-VP* transcription activator, and *GD-AtAUEs* or control *GD* were co-transfected into Arabidopsis protoplasts. Following 22 h of dark incubation, GUS activity was assayed with a microplate reader. Data are presented as mean ± SD of three biological replicates. Significantly different from that of the *GD* (*** *p* < 0.001).

We examined the subcellular localization of *AtAUEs* using Arabidopsis protoplast transfection assays. Constructs of *GFP-AtAUEs* were generated and co-transfected with an *NLS-RFP* nuclear indicator plasmid into isolated Arabidopsis protoplasts [42]. After 22 h of incubation, GFP and RFP fluorescence was analyzed using a fluorescence microscope. As depicted in Figure 3a, the overlap of GFP fluorescence with RFP suggests that *AtAUEs* were predominantly localized within the nucleus, though some fluorescence was also detected in other cellular regions, including the cell membranes.

We then assessed the transcriptional activity of *AtAUEs* on the *LexA-Gal4:GUS* reporter gene, which is activated by the *LD-VP* transcriptional activator [43]. The constructs of *GD-AtAUEs* were co-transfected with the *LexA-Gal4:GUS* reporter and *LD-VP* into isolated protoplasts of Arabidopsis. Co-transfection of the *GD* construct with *LexA-Gal4:GUS* and *LD-VP* served as a control. As illustrated in Figure 3b, a notable decrease in GUS activity was observed in protoplasts that were co-transfected with *AtAUEs*, indicating that *AtAUEs* repress the expression of the *LexA-Gal4:GUS* reporter gene activated by *LD-VP*.

### 2.3. ABA Responses Are Not Affected in the Ataue Single Mutants

After confirming that *AtAUEs* are ABA-responsive genes and that *AtAUEs* have transcriptional repression activities, to investigate their potential role in regulating the ABA responses in Arabidopsis, we generated *CRISPR/Cas9* gene-edited single mutants for the *AtAUE* gene clade, which includes *AtAUE1*, *AtAUE2*, and *AtAUE3*. This was accomplished by transforming Col wild-type Arabidopsis with *pHEE-FT* constructs containing specific target sequences. For each *AtAUE* gene, two distinct target sequences were chosen to create the constructs. In the T2 generation, Cas9-free homozygous mutants were identified based on their flowering phenotypes, PCR amplification results, and sequencing of the corresponding *AtAUE* genes and Cas9 fragment. For each of the three *AtAUE* genes, two single mutants (*ataue1-c1*, *ataue1-c2*, *ataue2-c1*, *ataue3-c1*, and *ataue3-c2*) were obtained for further experiments.

**Figure 4:**
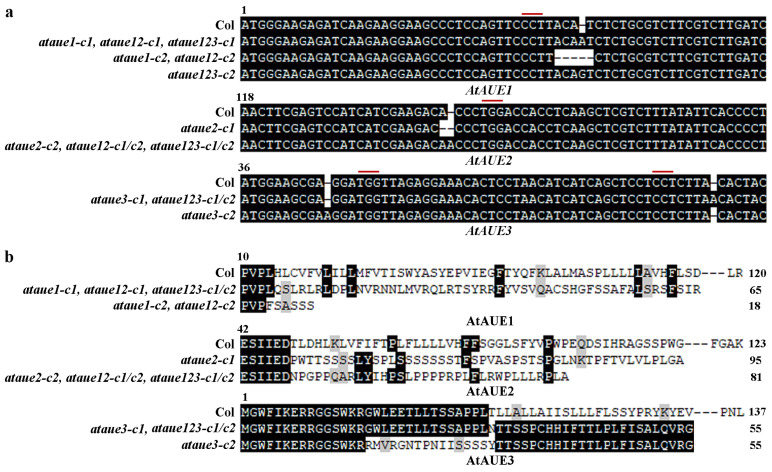
Generation of *ataue1*, *ataue2*, *ataue3* single mutants, *ataue12* double mutants, and *ataue123* triple mutants. (**a**) DNA sequence alignment of *AtAUE1*, *AtAUE2*, and *AtAUE3* in Col wild-type and the gene-edited *ataue1*, *ataue2*, and *ataue3* single mutants, as well as *ataue12* double and *ataue123* triple mutants. Numbers above the sequences indicate nucleotide positions relative to the start codon. Red lines indicate PAM sites. (**b**) Amino acid sequence alignment of AtAUE1, AtAUE2, and AtAUE3 in Col wild-type and the gene-edited mutants. The DNA sequences were analyzed for open reading frames (ORF) using ORFfinder (https://www.ncbi.nlm.nih.gov/orffinder/, accessed on 11 October 2017), and predicted amino acid sequences were aligned with those in Col wild-type. Numbers above the sequences indicate amino acid positions relative to the initiating methionine, and numbers at the end indicate total amino acid counts for the AtAUE proteins.

In the *ataue1* and *ataue2* single mutants, only one target sequence was edited, resulting in either a single nucleotide insertion or deletion or a four-nucleotide deletion at the target site (Figure 4a). In contrast, *ataue3* single mutants showed edits in both target sequences, with a single nucleotide insertion occurring in one target sequence for one mutant and in another target sequence for a different mutant (Figure 4a). All single mutants displayed nucleotide insertions/deletions, leading to amino acid substitutions and premature stop codons in the AtAUE proteins (Figure 4b).

To assess ABA responses in the generated single mutants, we performed three typical assays: ABA-inhibited seed germination, cotyledon greening, and root elongation.

**Figure 5:**
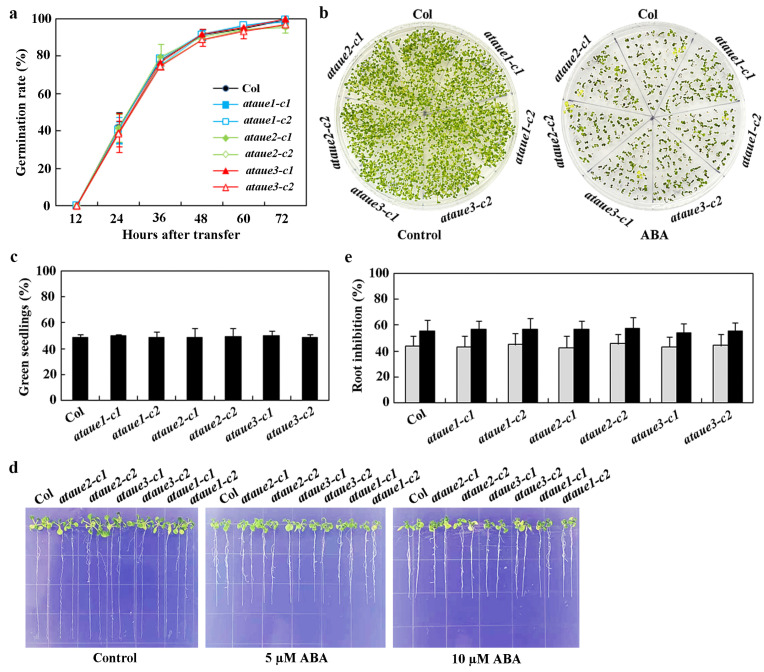
Effects of ABA on seed germination, cotyledon greening, and root elongation in Col wild-type and *ataue1*, *ataue2*, and *ataue3* single mutants. (**a**) Seed germination assay. Surface-sterilized seeds of Col wild-type and the single mutants were plated on 1/2 MS plates with or without 1 μM ABA. Plates were kept at 4 °C in the dark for 2 days, then transferred to a growth room. Germinated seeds were counted every 12 h, and germination percentages were calculated. Data are presented as mean ± SD of three replicates. (**b**) Cotyledon greening assay. Surface-sterilized seeds were plated as above. Images were taken 10 days after transfer to the growth room. (**c**) Quantitative analysis of green seedlings. The number of seedlings with green cotyledons was counted, and percentages were calculated. Data are presented as mean ± SD of three replicates. (**d**) Root elongation assay. Surface-sterilized seeds were plated on 1/2 MS plates, kept at 4 °C in the dark for 2 days, then grown vertically for 3 days in a growth room. Seedlings were transferred to control plates or plates containing 5 μM and 10 μM ABA for 7 additional days before imaging. (**e**) Quantitative analysis of root elongation inhibition by ABA. Root lengths of new elongation were measured after imaging, and the percentage of inhibition was calculated for ABA concentrations of 5 μM (gray) and 10 μM (black). Data are presented as means ± SD of 11–16 seedlings.

In seed germination assays, no significant differences were observed between the single mutants and Col wild-type (Figure 5a). Similarly, cotyledon greening assays showed no notable differences between the single mutants and Col wild-type (Figure 5b), a finding further confirmed by quantitative analysis of green seedlings (Figure 5c).

In root elongation assays, root lengths of all single mutants were comparable to those of Col wild-type in the absence of ABA. While ABA inhibited root elongation in both Col wild-type and single mutants, no differences were observed between them (Figure 5d). Quantitative analysis revealed that ABA significantly inhibited root elongation across all seedlings, with increasing inhibitory effects at higher ABA concentrations; however, no differences were noted between the single mutants and Col wild-type (Figure 5e).

### 2.4. ABA Responses Are Affected in the ataue123 Triple Mutants and Transgenic Plants Overexpressing AtAUE1

The above results indicate that ABA responses in Arabidopsis were not affected when a single *AtAUE* gene, including *AtAUE1*, *AtAUE2*, or *AtAUE3*, was knocked out. Considering that all *AtAUE* genes are ABA-responsive genes (Figure 1), *AtAUEs* are closely related proteins (Figure 2), and all have transcriptional repression activity (Figure 3), we assumed that *AtAUEs* may have a redundant function. To investigate whether this held true, we decided to generate CRISPR/Cas9 gene-edited *ataue1 ataue2* (*ataue12*) double and *ataue1 ataue2 ataue3* (*ataue123*) triple mutants and examined their responses to ABA treatment.

The *ataue12* double mutants were generated by editing *AtAUE1* in the transgene-free *ataue2-c2* single mutant plants, and the *ataue123* triple mutants were generated by editing *AtAUE1* in the transgene-free *ataue23* double mutant plants. In the *ataue12* double mutants, either a single nucleotide insertion or the deletion of four nucleotides occurred at one of the target sites of the *AtAUE1* gene (Figure 4a), which resulted in an amino acid substitution and a premature stop codon in the AtAUE1 protein (Figure 4b). In the *ataue123* triple mutants, either a single A or G nucleotide insertion occurred at one of the target sites of the *AtAUE1* gene (Figure 4a), which resulted in an amino acid substitution and a premature stop in the AtAUE1 protein (Figure 4b).

ABA-inhibited seed germination, cotyledon greening, and root elongation assays were utilized to examine the ABA responses of the *ataue12* double and *ataue123* triple mutants. In all assays, altered ABA responses were observed in the *ataue123* triple mutants but not in the *ataue12* double mutants, compared to the Col wild-type.

**Figure 6:**
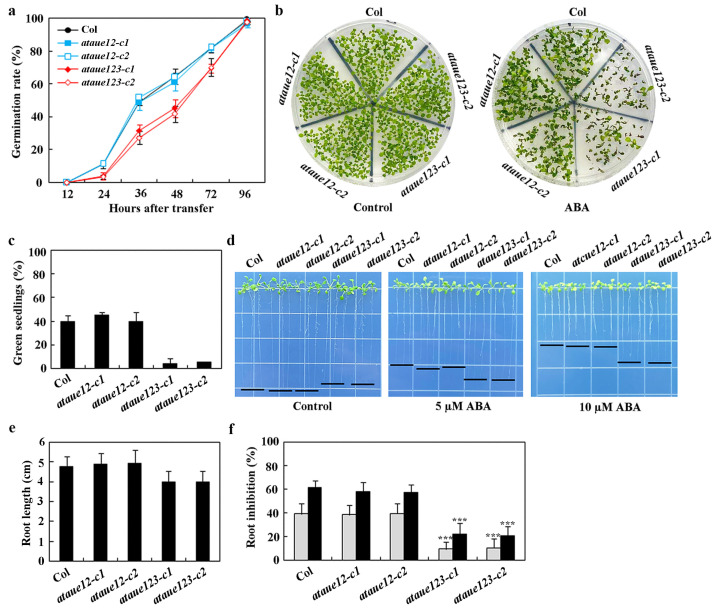
Effects of ABA on seed germination, cotyledon greening, and root elongation in Col wild-type, *ataue12* double mutants, and *ataue123* triple mutants. (**a**) Seed germination assay. Surface-sterilized seeds of Col wild-type, *ataue12* double mutants, and *ataue123* triple mutants were plated on 1/2 MS plates with or without 1 μM ABA. Plates were kept at 4 °C in the dark for 2 days, then transferred to a growth room. The number of germinated seeds was counted at the indicated times, and germination percentages were calculated. Data are presented as mean ± SD of three replicates. (**b**) Cotyledon greening assay. Surface-sterilized seeds were plated as above. Images were taken 15 days after transfer to the growth room. (**c**) Quantitative analysis of green seedlings. The number of seedlings with green cotyledons was counted, and percentages were calculated. Data are presented as mean ± SD of three replicates. (**d**) Root elongation assay. Surface-sterilized seeds were plated on 1/2 MS plates, kept at 4 °C in the dark for 2 days, then grown vertically for 3 days in a growth room. Seedlings were transferred to control plates or plates containing 5 μM and 10 μM ABA and grown for an additional 7 days before imaging. (**e**) Root length measurements. Root lengths of seedlings were measured after imaging. Data are presented as means ± SD of 26–38 seedlings. (**f**) Quantitative analysis of root elongation inhibition by ABA. Root lengths of newly elongated roots were measured after imaging, and the percentage of inhibition was calculated for ABA concentrations of 5 μM (gray) and 10 μM (black). Data are presented as means ± SD of 26-38 seedlings. Significantly different from that of the Col wild-type seedlings (*** *p* < 0.001).

In seed germination assays, the *ataue12* double mutants exhibited a similar ABA response to the Col wild-type, while significant reductions in germination were noted for seeds of the *ataue123* triple mutants (Figure 6a). A comparable result was observed in the cotyledon greening assays, where the *ataue12* double mutants and the Col wild-type produced a similar number of green seedlings in the presence of ABA, whereas the number of green seedlings in the *ataue123* triple mutants was greatly diminished (Figure 6b). Quantitative results indicated that both the Col wild-type and the *ataue12* double mutants had approximately a 40% green seedling rate in the presence of ABA, while the *ataue123* triple mutants showed only about a 5% green seedling rate (Figure 6c).

In the root elongation assays, we observed that the roots of the *ataue123* triple mutants were significantly shorter than those of the Col wild-type and the *ataue12* double mutants on control plates (Figure 6d). The root lengths measured approximately 5 cm for both the Col and *ataue12* double mutants, while the *ataue123* triple mutants had a length of only about 4 cm (Figure 6e). Notably, the *ataue123* triple mutants exhibited a distinct ABA response, producing longer roots than both the Col wild-type and *ataue12* double mutants at all concentrations tested (Figure 6d).

Quantitative results indicated that, in the presence of 5 µM ABA, there was approximately a 40% inhibition in both the Col wild-type and *ataue12* double mutants, whereas the *ataue123* triple mutants showed only about 10% inhibition. With 10 µM ABA, the inhibition increased to roughly 60% for the Col wild-type and *ataue12* double mutants but only to about 20% for the *ataue123* triple mutants (Figure 6f). These findings suggest that *AtAUE1*, *AtAUE2*, and *AtAUE3* function redundantly in regulating ABA responses in Arabidopsis.

To further investigate their roles, we generated transgenic plants overexpressing *AtAUE1* by transforming Col wild-type with *pPZP211-35S:AtAUE1* and selected homozygous lines in the T3 generation. We assessed ABA responses using assays for ABA-inhibited seed germination, cotyledon greening, and root elongation. The *35S:AtAUE1* transgenic plants exhibited ABA responses opposite to those of the *ataue123* triple mutants in all assays.

**Figure 7:**
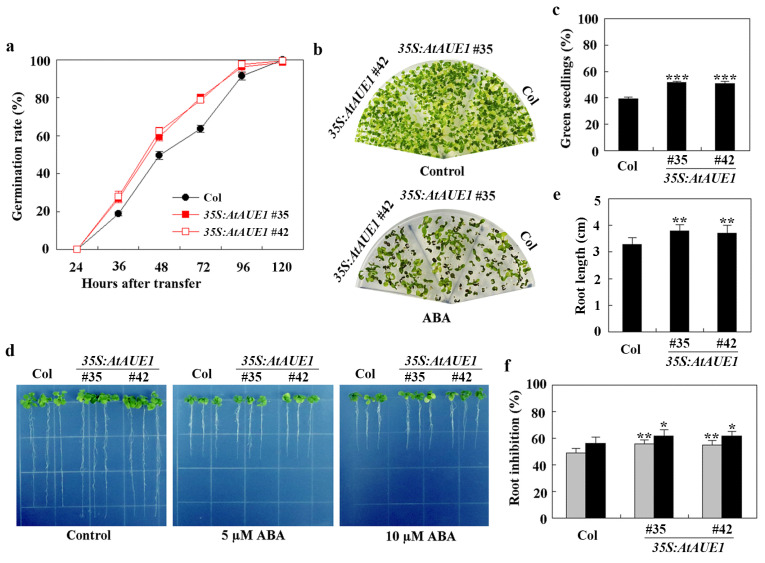
Effects of ABA on seed germination, cotyledon greening, and root elongation of Col wild-type and *35S:AtAUE1* transgenic plants. (**a**) Seed germination assay. Surface-sterilized seeds of Col wild-type and *35S:AtAUE1* transgenic plants were plated on 1/2 MS plates with or without 1 μM ABA. Plates were kept at 4 °C in the dark for 2 days, then transferred to a growth room. The number of germinated seeds was counted at specified time points, and the percentage of germination was calculated. Data are presented as mean ± SD of three replicates. (**b**) Cotyledon greening assay. Surface-sterilized seeds were plated on 1/2 MS plates with or without 1 μM ABA. After a 2-day incubation at 4 °C in the dark, plates were transferred to a growth room. Photographs were taken 15 days after the transfer. (**c**) Quantitative analysis of green seedlings. The number of seedlings with green cotyledons was counted from the photographs, and the percentage of green cotyledons was calculated. Data are presented as mean ± SD of three replicates. (**d**) Root elongation response to ABA. Surface-sterilized seeds were plated on 1/2 MS plates. Plates were kept at 4 °C in the dark for 2 days, then transferred to a growth room for vertical growth over 3 days. Seedlings were subsequently transferred to control plates and plates containing 5 μM or 10 μM ABA, and grown for an additional 5 days before photographs were taken. (**e**) Root length measurement. Root lengths of seedlings were measured post-photographing. Data are shown as mean ± SD of 12–16 seedlings. (**f**) Quantitative analysis of root elongation inhibition by ABA. The lengths of newly elongated roots were measured after photographing, and the percentage of inhibition was calculated for 5 μM (gray) and 10 μM (black) ABA treatments. Data are presented as mean ± SD of 12–16 seedlings. Significantly different from that of the Col wild-type seedlings (* *p* < 0.05, ** *p* < 0.01, *** *p* < 0.001).

In seed germination tests, the germination rate of *35S:AtAUE1* plants was higher than that of Col wild-type in the presence of ABA (Figure 7a). In cotyledon greening assays, *35S:AtAUE1* plants produced more green seedlings than Col wild-type when ABA was present (Figure 7b), with approximately 40% green seedlings for Col wild-type and about 50% for *35S:AtAUE1* (Figure 7c).

In root elongation assays, *35S:AtAUE1* plants showed longer roots on control plates, measuring around 4 cm compared to about 3 cm for Col wild-type (Figure 7d,e). However, in the presence of ABA, root lengths of *35S:AtAUE1* plants were similar to those of Col wild-type, indicating increased ABA sensitivity. Quantitative results confirmed that, with 5 µM and 10 µM ABA, there was an approximate 5% increase in the root elongation inhibition in *35S:AtAUE1* plants compared to Col wild-type (Figure 7f).

### 2.5. Expression of ABI5 Was Increased in the ataue123 Triple Mutants but Decreased in the Transgenic Plants Overexpressing AtAUE1

**Figure 8:**
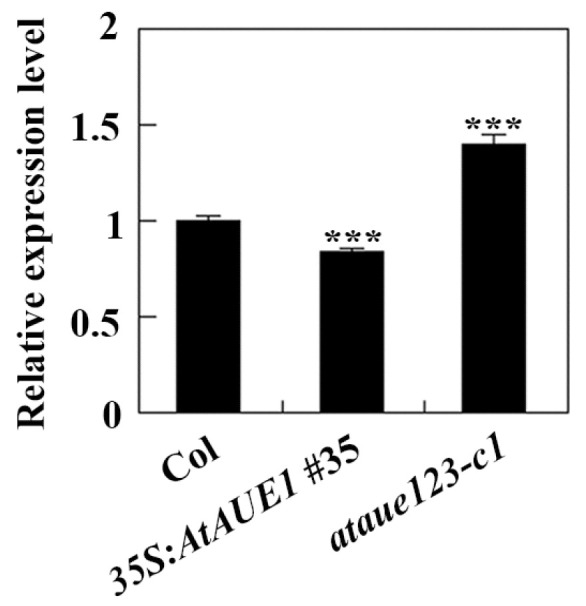
Expression of *ABI5* in Col wild-type, *35S:AtAUE1* transgenic plants, and *ataue123* triple mutants. Total RNA was isolated from ten-day-old seedlings of Col wild-type, *35S:AtAUE1* transgenic plants, and *ataue123* triple mutants. cDNA was synthesized and used for qRT-PCR to assess *ABI5* expression, with *ACT2* serving as an internal control. *ABI5* expression levels in Col wild-type seedlings were normalized to 1, and relative expression levels in *35S:AtAUE1* and *ataue123* triple mutants were calculated accordingly. Data are presented as mean ± SD of three technical replicates. Significantly different from that of the Col wild-type (*** *p* < 0.001).

The results suggest that *AtAUEs* exhibit functional redundancy in regulating ABA responses in Arabidopsis. To explore their potential mechanisms, we examined the expression of key ABA-signaling regulator genes in Col wild-type, *35S:AtAUE1* transgenic plants, and *ataue123* triple mutants using qRT-PCR. This analysis aimed to determine whether *AtAUEs* influence ABA signaling and thereby affect ABA responses. Given that *AtAUEs* can repress co-transfected reporter gene expressions (Figure 3) and that the *ataue123* triple mutants and *35S:AtAUE1* plants exhibit opposite responses to ABA treatments (Figure 6 and Figure 7), we focused on genes with contrasting expression patterns between these plant types. Notably, the expression level of *ABI5*, a key regulator in ABA signaling, was increased in *ataue123* triple mutants but decreased in *35S:AtAUE1* transgenic plants (Figure 8).

## 3. Discussion

Abscisic acid (ABA), acting as a stress hormone, triggers the activation of ABA-response genes via signaling transduction pathways, ultimately regulating plant responses to both ABA and environmental stresses [4,6,7,8,9]. Through the characterization of previously unknown functions of ABA-response genes, we have discovered several novel regulators of ABA responses, encompassing AITRs, AtEAUs, ASDs, and ASRs [10,23,25,36,39]. Notably, some of these regulators are proteins that contain the EAR motif [10,23,25,36]. In this study, we provide evidence that *AtAUEs* constitute a novel small family of EAR motif-containing regulators involved in ABA responses, exhibiting redundant functions in regulating ABA responses in Arabidopsis.

First, the expression of *AtAUEs* is up-regulated by ABA, and *AtAUEs* function as transcription repressors. From the previous transcriptome dataset, we identified *AtAUEs* as functional uncharacterized ABA-responsive genes, and their up-regulation by ABA was further confirmed by qRT-PCR (Figure 1). Amino acid sequence alignments show that *AtAUEs* are EAR motif-containing proteins, with *AtAUE2* containing two LxLxL EAR motifs, while the other four *AtAUEs* have only one LxLxL EAR motif (Figure 2). Consistent with a previous observation that EAR motifs are responsible for transcriptional repression of the repressors identified, such as ERF repressors, Aux/IAA proteins, and some of the C2H2 transcription factors [27,28,34,42], *AtAUEs* repressed the expression of a co-transfected reporter gene in Arabidopsis protoplasts, even though *AtAUEs* were not predominately localized in the nucleus (Figure 3), suggesting that *AtAUEs* may function as transcription repressors. One possible explanation is that *AtAUEs* may be transported outside the nucleus through interactions with other proteins or RNAs. These interactions may involve specific amino acid residues that play a crucial role in the extranuclear localization of *AtAUEs*.

Second, we found that *AtAUEs* regulate ABA responses in Arabidopsis. Even though the single mutants *ataue1*, *ataue2*, and *ataue3*, as well as the double mutants *ataue12*, are largely similar to the Col wild-type in ABA-response assays (Figure 5 and Figure 6), rates of seed germination and green seedling and the percentage of root inhibition were decreased in the *ataue123* triple mutants in the presence of ABA (Figure 6). Conversely, the *35S:AtAUE1* transgenic plants displayed an increased percentage of seed germination, green seedling emergence, and root inhibition in response to ABA treatment (Figure 7). These results suggest that *AtAUEs* are involved in the regulation of ABA responses in Arabidopsis and that they have redundant functions. Third, the expression level of *ABI5*, one of the ABA-signaling key transcriptional regulator genes [16,17], was increased in the *ataue123* triple mutants but decreased in the *35S:AtAUE1* transgenic plants (Figure 8); ABI5 is a key component in the ABA-signaling pathway, functioning as a transcriptional factor that plays a central role in the response to ABA. When ABA is present, a series of signal transduction events occur, ultimately leading to the up-regulation of ABI5 expression. The up-regulation of ABI5 further regulates the expression of downstream genes, thereby modulating the plant’s response to ABA, such as seed dormancy, stomatal closure, and stress tolerance. Our results indicated that *AtAUEs* might play a feedback-regulating role in ABA signaling and, therefore, regulate ABA responses in Arabidopsis.

It should be noted that *AtAUEs* may play distinct roles in regulating plant ABA responses across different tissues or growth stages. In the presence of ABA, the *ataue123* triple mutants showed reduced rates of seed germination and green seedling growth, whereas the *35S:AtAUE1* transgenic plants exhibited increased rates of both. (Figure 6 and Figure 7), suggesting that *AtAUEs* negatively regulate ABA sensitivity during these processes. Conversely, root inhibition percentage decreased in the *ataue123* mutants and increased in the *35S:AtAUE1* transgenic plants with ABA treatment (Figure 6 and Figure 7), indicating that *AtAUEs* positively regulate ABA sensitivity in root elongation. Given that all *AtAUEs* inhibited the expression of co-transfected reporter genes in Arabidopsis protoplasts, it is likely that *AtAUEs* interact with different proteins depending on tissue or growth stage, thus differentially regulating ABA responses. It would be intriguing to explore this possibility further. Additionally, while our results show that *ABI5* expression was increased in the *ataue123* triple mutants and decreased in the *35S:AtAUE1* plants (Figure 8), further investigation is required to determine if *AtAUEs* differentially regulate *ABI5* expression across various tissues or growth stages whether *ABI5* is a direct target gene of *AtAUEs*, and also to identify other genes that may be influenced by *AtAUEs*.

It is well known that the regulation of ABA-response genes via ABA signaling will eventually affect plant abiotic stress responses [3,4,7,9,17,18,19,20]. Since ABA sensitivity was altered in the *ataue123* triple mutants and the *35S:AtAUE1* transgenic plants in a tissue/organ or growth stage-dependent manner (Figure 6 and Figure 7), it is very likely that *AtAUEs* may regulate plant abiotic stress responses. Future experiments need to be carried out to examine if this is indeed the case and if *AtAUEs* may regulate plant abiotic stress responses in a tissue/organ or growth stage-dependent manner.

In addition to exhibiting altered sensitivity to ABA, we noted that the root length of the *ataue123* triple mutants decreased, whereas it increased in the *35S:AtAUE1* transgenic plants. In contrast, root lengths of the *ataue1*, *ataue2*, and *ataue3* single mutants, as well as the *ataue12* double mutants, were largely similar to that of the Col wild-type (Figure 5, Figure 6 and Figure 7), indicating that *AtAUEs* may function redundantly to regulate root elongation in Arabidopsis. Therefore, it is worthwhile to investigate how *AtAUEs* regulate root elongation and whether this regulation is dependent on ABA signaling.

Considering that it has been shown that the expression of *AtAUE4* is affected by growth and development regulators ANAC012 and the Retarded Growth of Embryo1 (RGE1) [43,44], examining if *AtAUEs* regulate other aspects of plant growth and development may also be enlightening.

## 4. Materials and Methods

### 4.1. Plant Materials and Growth Conditions

The Columbia-0 (Col) ecotype of Arabidopsis was used to investigate the expression of *AtAUE* genes in response to ABA treatment, for protoplast isolation to assess the subcellular localization and transcriptional activity of *AtAUEs*, and for generating all mutants and transgenic lines used in this study, serving as a wild-type control for analyzing ABA-inhibited seed germination, cotyledon greening, and root elongation. *AtAUE1* overexpression plants were produced by transforming Col wild-type plants with the *35S:AtAUE1* construct. The *ataue1*, *ataue2*, and *ataue3* single mutants, as well as the *ataue12* double and *ataue123* triple mutants, were generated through CRISPR/Cas9 editing of the corresponding *AtAUE* genes in Col wild-type plants.

For protoplast isolation and plant transformation, Col wild-type seeds were soaked in water at 4 °C for 2 days, then sown directly into soil pots and grown in a growth room. For RNA isolation and ABA-response assays, seeds of Col wild-type, transgenic plants, and Cas9-free mutants were sterilized in 25% (*v*/*v*) bleach for 10 min, washed four times with sterilized water, and plated on 1/2 MS (Murashige and Skoog) plates supplemented with vitamins and 1% (*w*/*v*) sucrose, solidified with 0.6% (*w*/*v*) phytoagar. Plates were kept at 4 °C for 2 days before transfer to the growth room.

Growth conditions were maintained at 22 °C with a photon density of approximately 120 μmol m^−2^ s^−1^ under a photoperiod of 16 h light and 8 h dark.

### 4.2. ABA Treatment, RNA Isolation, RT-PCR, and qRT-PCR

To examine the expression of *AtAUEs* in response to ABA treatment, seeds of Col wild-type plants were germinated and grown on 1/2 MS plates for 10 days. The seedlings were then treated with 50 μM ABA in the dark for 4 h. For assessing *ABI5* expression in *35S:AtAUE1* overexpression plants and *ataue123* triple mutants, seeds of Col wild-type, *35S:AtAUE1* plants, and *ataue123* mutants were similarly germinated and grown for 10 days. Seedlings were collected and frozen in liquid nitrogen, and RNA was isolated. cDNA was synthesized and used to detect *AtAUEs* and *ABI5* expression following previously described procedures [45,46]. *ACTIN2* (*ACT2*) served as an internal control gene for quantitative RT-PCR analysis; primers for *ABI5* and *ACT2* have been reported [45,46], while primers for *AtAUEs* are listed in Table S1. Gene expression levels were analyzed using the 2^−ΔΔCT^ method, with three biological replicates conducted for each experiment.

### 4.3. Constructs

The *NLS-RFP* construct served as a nuclear indicator [41], while the *LexA-Gal4:GUS* reporter construct, *LD-VP* activator construct, and *GD* control construct were used for protoplast transfection to assess transcriptional repressor activities [37,47]. To generate the *GD-AtAUEs* and *GFP-AtAUEs* constructs for protoplast transient transfection, the full-length open reading frame (ORF) of *AtAUEs* was amplified by RT-PCR using RNA from 10-day-old Col wild-type seedlings. The amplified product was double digested with *NdeI* and *SacI*, then cloned in-frame with an N-terminal *GD* or *GFP* tag into the *pUC19* vector under the *CaMV 35S* promoter.

For the *35S:AtAUE1* construct for plant transformation, the *NdeI* and *SacI*-digested AtAUE1 was cloned in-frame with an N-terminal HA tag into the *pUC19* vector, also under the *CaMV 35S* promoter. This construct was then digested with *PstI* and *SacI* and cloned into the binary vector *pPZP211* [48].

To create CRISPR/Cas9 constructs for the gene editing of *AtAUE1*, *AtAUE2*, and *AtAUE3*, the genomic sequences of *AtAUEs* were analyzed using CRISPRscan (http://www.crisprscan.org, accessed on 12 April 2018) to identify potential target sequences. Selected sequences were further evaluated with Cas-OFFinder (http://www.rgenome.net/cas-offinder/, accessed on 12 April 2018) to assess potential off-target effects. The target sequences selected for *AtAUE1* are (CCT)TACATCTCTGCGTCTTCGTC and GAGCTGGAGGAACACCGTGG(GGG), for *AtAUE2* are (CCT)CTCTCTCCCAAACCCACATC and GTCCATCATCGAAGACACCC(TGG), and *AtAUE3* are GAGGTTCATGGAAGCGAGGA(TGG) and (CCT)CTTACACTACTAGCTCTCCT. The selected target sequences were inserted into the *pHEE401E-FT* vector using the procedure described previously [49]. The primers utilized to generate the *pHEE401E-FT-AtAUE1*, *pHEE401E-FT-AtAUE2*, and *pHEE401E-FT-AtAUE3* constructs are listed in Table S1.

### 4.4. Plant Transformation, Transgenic Plant Selection, and Transgene-Free Mutant Isolation

To generate overexpression plants and transgene-free single mutants, approximately 5-week-old Col wild-type plants with several mature flowers were transformed with the *pPZP-35S:AtAUE1*, *pHEE401E-FT-AtAUE1*, *pHEE401E-FT-AtAUE2*, and *pHEE401E-FT-AtAUE3* constructs using the floral dip method [50]. The isolated transgene-free *ataue2-c2* single mutant plants were subsequently transformed with the *pHEE401E-FT-AtAUE1* construct to produce *ataue12* double mutants. Additionally, the transgene-free *ataue23* double mutant plants were transformed with the *pHEE401E-FT-AtAUE1* construct to generate *ataue123* triple mutants.

T1 seeds were germinated on antibiotic-containing 1/2 MS plates to identify transgenic plants. To isolate homozygous transgenic lines, T2 seeds from *35S:AtAUE1* transgenic plants were germinated on antibiotic-containing 1/2 MS plates to select lines with a single T-DNA insertion. Homozygous lines were confirmed by germinating T3 seeds on similar plates. Two homozygous transgenic lines with high *AtAUE1* expression were used for experiments. To isolate homozygous transgene-free gene-edited mutants, T2 seeds from early flowering T1 plants were germinated directly in soil pots to identify normal flowering, transgene-free plants. The gene-editing status of these T2 transgene-free plants was examined by amplifying and sequencing the genomic sequences of *AtAUE1*, *AtAUE2*, or *AtAUE3* to identify homozygous mutants. The *Cas9* gene fragment was confirmed by PCR amplification for detecting transgene-free status in these homozygous mutants [49].

### 4.5. DNA Isolation and PCR

To examine the editing status of *AtAUE1*, *AtAUE2*, and *AtAUE3*, DNA was isolated from the leaves of early flowering T1 plants or normal flowering T2 transgenic plants and used as a template for PCR amplification and sequencing. To isolate transgene-free mutants, DNA was extracted from the leaves of normal flowering T2 transgenic plants and used for PCR amplification of the *Cas9* gene fragment. The primers employed for amplifying the *Cas9* fragment have been described previously [49].

### 4.6. Plasmid DNA Isolation, Protoplast Isolation, and Transfection

Plasmid DNA of the reporter and effector constructs was isolated using the Endo-Free Plasmid Maxi Kit (OMEGA) according to the manufacturer’s instructions. Arabidopsis protoplasts were isolated from the rosette leaves of 3- to 4-week-old Col wild-type plants and transfected as previously described [10,45,51].

For the subcellular localization assay, plasmids of *GFP-AtAUEs* and *NLS-RFP* were co-transfected into the isolated protoplasts, which were incubated in the dark for 22 h at room temperature. GFP and RFP fluorescence was observed using an Olympus FV1000 confocal microscope.

For the transcriptional activity assay, the reporter gene plasmid *LexA-Gal4:GUS*, the activator gene *LD-VP*, and the effector gene *GD-AtAUEs* or control gene *GD* were co-transfected into isolated protoplasts, incubated in the dark for 22 h, and then GUS activities were measured using a Synergy™ HT microplate reader.

### 4.7. ABA Sensitivity Assays

ABA-inhibited seed germination, cotyledon greening, and root elongation assays were conducted as previously described [52,53]. Seeds of Col wild-type, *35S:AtAUE1* transgenic plants and transgene-free mutants were sterilized and plated on 1/2 MS plates containing 1 μM ABA or on control plates without ABA. The plates were kept in the dark at 4 °C for 2 days before being transferred to a growth room. Germinated seeds were counted at specified time points, and the percentage of germination was calculated. Photographs were taken to count seedlings with green cotyledons, allowing for the calculation of the percentage of green seedlings. At least 45 seeds per genotype were used, and experiments were repeated three times.

For ABA-inhibited root elongation assays, Surface-sterilized seeds of Col wild-type and *35S:AtAUE1* transgenic plants or transgene-free mutants were plated on 1/2 MS plates. After incubation in the dark at 4 °C for 2 days, the plates were transferred to a growth room for vertical germination. After 3 days, germinated seeds were selected and transferred to 1/2 MS plates containing 0, 5, and 10 μM ABA for vertical growth. Root lengths and/or lengths of newly elongated roots were measured at specified time points, and the percentage of inhibition was calculated. At least 11 seedlings per genotype were used, and this experiment was repeated three times.

## Data Availability

All data are presented in the manuscript.

## Supplementary Materials

The Supplementary Materials are available online at https://www.mdpi.com/article/10.3390/plants13233282/s1, Table S1: Primers used in this study.
