# Supplementary material for: AtAUEs, a Small Family of ABA Up-Regulated EAR Motif-Containing Proteins Regulate ABA Responses in Arabidopsis
Source: Plants (Basel). 2024 Nov 22;13(23):3282. doi: 10.3390/plants13233282 (PMC11644342; doi:10.3390/plants13233282)
Supplement: Supplementary file 1 [file plants-13-03282-s001.zip › plants-3257140-supplementary.pdf]

Table S1. Primers used in this study

Primers	Sequences (5'--3')
AtAUE1-qF	TCAAGCTTGCTCTCATGGCT
AtAUE1-qR	GACGTCATCATCCCTCCGAC
AtAUE2-qF	CATCGAAGACACCCTGGACC
AtAUE2-qR	CCAGGGGACGTAGAAGGAGA
AtAUE3-qF	CTTCCTCTCTTCATATCCGCGTTA
AtAUE3-qR	TGGACGAAGCTTAGAGAGACG
AtAUE4-qF	CGAATCAGCGGAAGAGCAGA
AtAUE4-qR	AGAAGCGCATCAGGGTTCTC
AtAUE5-qF	TCCTTCGTGTCGGCCATTTT
AtAUE5-qR	GGAAGAGAGCAACACCCACG
DT1-BsF-AtAUE1	ATATATGGTCTCGATTGGCGAAGACGCAGAGATGTAGTT
DT1-F0-AtAUE1	TGGCGAAGACGCAGAGATGTAGTTTTAGAGCTAGAAATAGC
DT2-R0-AtAUE1	AACCACGGTGTTCCTCCAGCTCCAATCTCTTAGTCGACTCTAC
DT2-BsR-AtAUE1	ATTATTGGTCTCGAAACCACGGTGTTCCTCCAGCTCC
DT1-BsF-AtAUE2	ATATATGGTCTCGATTGGTGTGGGTTTGGGAGAGAGGTT
DT1-F0-AtAUE2	TGGTGTGGGTTTGGGAGAGAGGTTTTAGAGCTAGAAATAGC
DT2-R0-AtAUE2	AACTTGGGGCGTAGCAGCCGTCCAATCTCTTAGTCGACTCTAC
DT2-BsR-AtAUE2	ATTATTGGTCTCGAAACTTGGGGCGTAGCAGCCGTCC
DT1-BsF-AtAUE3	ATATATGGTCTCGATTGAGGTTCATGGAAGCGAGGAGTT
DT1-F0-AtAUE3	AGGTTCATGGAAGCGAGGAGTTTTAGAGCTAGAAATAGC
DT2-R0-AtAUE3	CTGCTAATCTCAAGCTCTTCAATCTCTTAGTCGACTCTAC
DT2-BsR-AtAUE3	ATTATTGGTCTCGAAACCTGCTAATCTCAAGCTCTTC
